# Automated Peritoneal Dialysis Is Associated with Better Survival Rates Compared to Continuous Ambulatory Peritoneal Dialysis: A Propensity Score Matching Analysis

**DOI:** 10.1371/journal.pone.0134047

**Published:** 2015-07-27

**Authors:** Gabriela de Carvalho Beduschi, Ana Elizabeth Figueiredo, Marcia Olandoski, Roberto Pecoits-Filho, Pasqual Barretti, Thyago Proenca de Moraes, on behalf of all centers that contributed to the BRAZPD

**Affiliations:** 1 School of Medicine, UNESP, Botucatu, Brazil; 2 Graduate Program in Medicine and Health Sciences, Pontifícia Universidade Católica do Rio Grande do Sul (PUCRS), Porto Alegre, Brazil; 3 School of Medicine, Pontifícia Universidade Católica do Paraná (PUCPR), Curitiba, Brazil; Hospital Universitario de La Princesa, SPAIN

## Abstract

**Introduction:**

The impact of peritoneal dialysis modality on patient survival and peritonitis rates is not fully understood, and no large-scale randomized clinical trial (RCT) is available. In the absence of a RCT, the use of an advanced matching procedure to reduce selection bias in large cohort studies may be the best approach. The aim of this study is to compare automated peritoneal dialysis (APD) and continuous ambulatory peritoneal dialysis (CAPD) according to peritonitis risk, technique failure and patient survival in a large nation-wide PD cohort

**Methods:**

This is a prospective cohort study that included all incident PD patients with at least 90 days of PD recruited in the BRAZPD study. All patients who were treated exclusively with either APD or CAPD were matched for 15 different covariates using a propensity score calculated with the nearest neighbor method. Clinical outcomes analyzed were overall mortality, technique failure and time to first peritonitis. For all analysis we also adjusted the curves for the presence of competing risks with the Fine and Gray analysis.

**Results:**

After the matching procedure, 2,890 patients were included in the analysis (1,445 in each group). Baseline characteristics were similar for all covariates including: age, diabetes, BMI, Center-experience, coronary artery disease, cancer, literacy, hypertension, race, previous HD, gender, pre-dialysis care, family income, peripheral artery disease and year of starting PD. Mortality rate was higher in CAPD patients (SHR1.44 CI95%1.21-1.71) compared to APD, but no difference was observed for technique failure (SHR0.83 CI95%0.69-1.02) nor for time till the first peritonitis episode (SHR0.96 CI95%0.93-1.11).

**Conclusion:**

In the first large PD cohort study with groups balanced for several covariates using propensity score matching, PD modality was not associated with differences in neither time to first peritonitis nor in technique failure. Nevertheless, patient survival was significantly better in APD patients.

## Introduction

The association between peritoneal dialysis (PD) modalities and clinical outcomes, namely peritonitis rates, technique failure and patient survival remains a controversial issue. First, the number of studies comparing the influence of modality on peritonitis rates is limited and only 3 studies presented a large sample size[1–3]. The initial results in favor of automated peritoneal dialysis (APD) have changed over time, and the APD benefit apparently disappears in studies published after the year of 2000. These changes may reflect improvements in the continuous ambulatory peritoneal dialysis (CAPD) connection systems[4]. On the other hand, the connection systems for APD have also improved throughout the time, but the majority of published comparison studies are based on patients treated during the nineties or early 2,000; only one recent large study compared peritonitis rates in APD and CAPD, reflecting the connection system used at the moment in both modalities[3].

Regarding technique survival, there is also no clear differences across PD modalities, with some data favoring APD[5–7], while other showing no differences between APD and CAPD [8,9]. Finally, information regarding patient survival relies vastly on observational studies, since no randomized controlled trial used mortality as an endpoint. Most of the observational trials found no significant differences between the PD modalities[10,11]. Three studies showed a beneficial effect of APD, two of them were single center and one a registry study[6,7,12]. In fact, a large RCT comparing hard clinical outcomes is unlikely to be available in the near future for several reasons, including the fact that the medical decision is usually driven by membrane characteristics and patient preference. In the absence of a RCT, the use of an advanced matching procedure to reduce selection bias in large cohort studies may be an adequate alternative for the comparative analysis between PD modalities.

Therefore, the aim of this study was to compare peritonitis risk, technique failure and patient survival between modalities in a large PD cohort, trying to minimize the effect of selection bias through an advanced matching technique for several covariates.

## Methods

This is an analysis of the nationwide prospective BRAZPD II cohort, previously described in details elsewhere[13]. We included all incident adult patients who remained at least 90 days in PD and excluded those who switched modality at any time during the follow-up period. Patients were recruited in 122 centers across the country and data was collected monthly from December 2004 to January 2011. The option not to include patients on PD for less than 90 days was taken to avoid the potential influence of prior therapies on clinical outcomes. To minimize the effects of the different comorbidities prevalence across the groups on clinical outcomes, we matched CAPD patients to individuals on APD using several covariates as described below. Hypertension was defined as according to the WHO/ISH criteria, a systolic blood pressure > 140mmHg and/or diastolic blood pressure > 90mmHg at baseline with or without use of hypertensive medication. The medical ethical committees of all participating centers approved the study. The list of all ethic review boards that approved the study can be found in the S5 Table. All patients provided written consent, which was approved by the ethical committee and stored locally only in Portuguese.

### Matching procedure

A set of covariates was selected to estimate the propensity score. These were: age, body mass index (BMI), center experience, Davies score, diabetes, family income, gender, literacy, PD modality, race, previous hemodialysis (HD), duration of pre-dialysis care and year of initiation of PD. The propensity score (PS) was calculated using logistic regression, as proposed by Fine and Gray [14], and CAPD patients were matched with APD controls using the nearest neighbor technique with a predefined caliper of 0.2. Groups were matched in a ratio of 1:1. This matching procedure was done using the MatchIt package for R[15].

### Clinical outcomes

Clinical outcomes were analyzed using both the traditional Cox Proportional Hazards model and adjusted for the presence of competing risk analysis as proposed by Fine and Gray [14]. For patient survival, the event of interest was death from any cause; for technique survival the event of interest was a defined as switch to HD for any cause, and for peritonitis risk the time to first peritonitis episode was the event of interest. Competing risks were defined as follows: (1) for mortality, any cause of drop out from therapy apart from death; (2) for technique failure, any cause of drop out from therapy apart from switching from PD to HD and (3) for time to first peritonitis any cause of drop-out occurred before the first episode of peritonitis. All patients still alive at the end of the study were treated as censored.

#### Sensitivity analysis

We performed a sensitivity analysis aiming to reduce the impact of lacking of data on residual renal function. We categorized our population in two groups: group I comprising patients with presumed RRF and group II with presumed no RRF. To be allocated in group I the patient should have at least one measured urinary volume > 100ml along the study (this represent in average 10% of our population) or be on diuretics, assuming that diuretics are only prescribed to patients with RRF. We then included this variable in the model after analyze its effect in univariate analysis.

### Statistical analysis

Continuous variables were expressed as mean ± SD or median and range, while categorical variables (e.g., gender, race, primary renal disease, presence of comorbid conditions, initial therapy, current PD modality, etc.) were expressed as frequencies or percentages. Data were analyzed using Student's t-test and the Chi-square test for categorical variables and ANOVA for comparison of continuous variables. Normality was checked using the Kolmogorov-Smirnov test. Cox proportional hazard models were estimated using SPSS 20.0 and sub-hazard distribution using competing risk analysis were calculated with the CRR function available in the CMPRSK package for R. For inclusion in the multivariate model, the covariate should have a p value lower than 0.20 in the univariate analysis. Collinearity was checked for all covariates potential covariates to be included in a model. Assumptions for proportional hazards and proportional sub-distribution hazards were checked with residual plots. Statistical significance was set at the level of p<0.05.

## Results

### Study population and baseline characteristics

**Fig 1:**
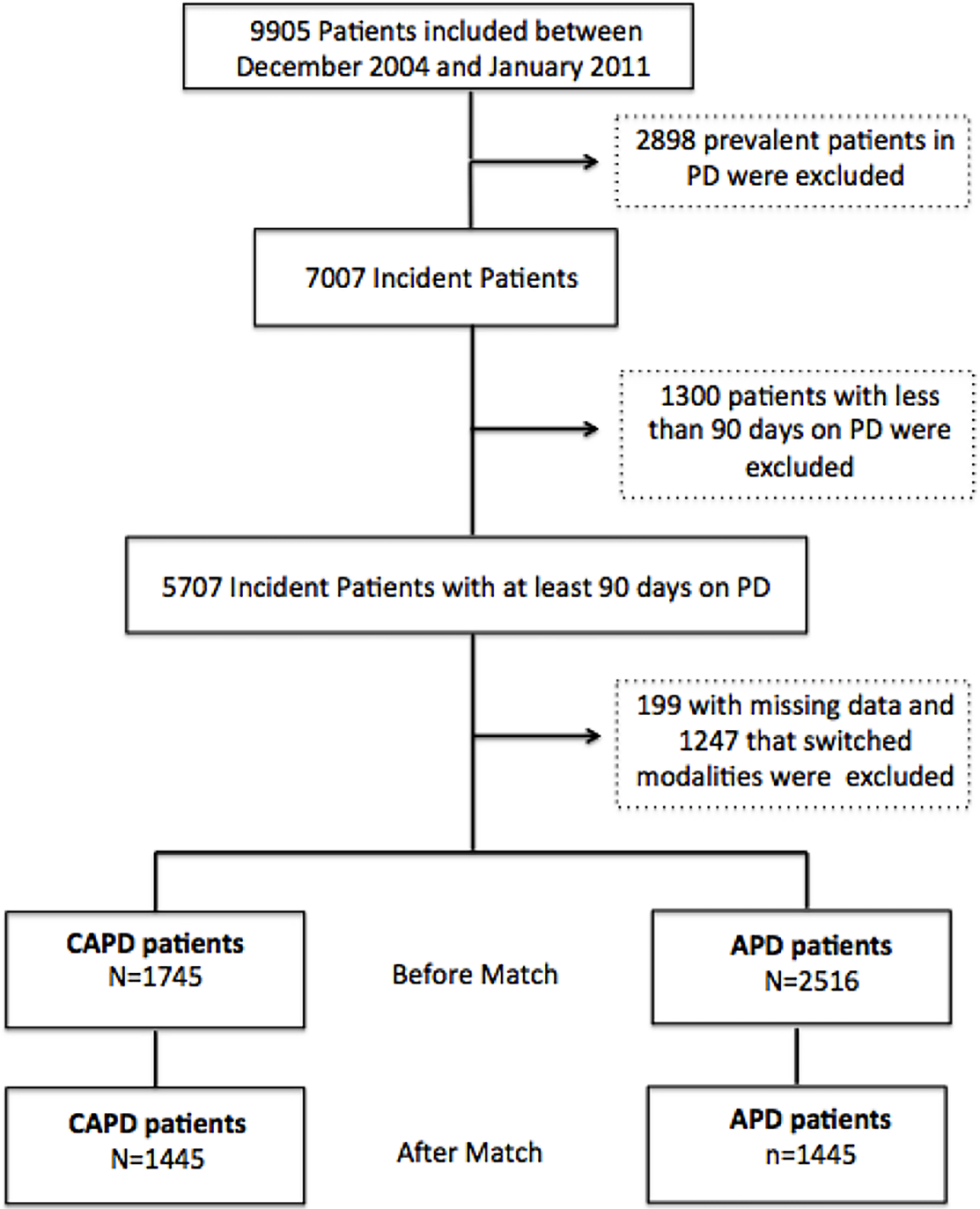
Study Population.

**Fig 2:**
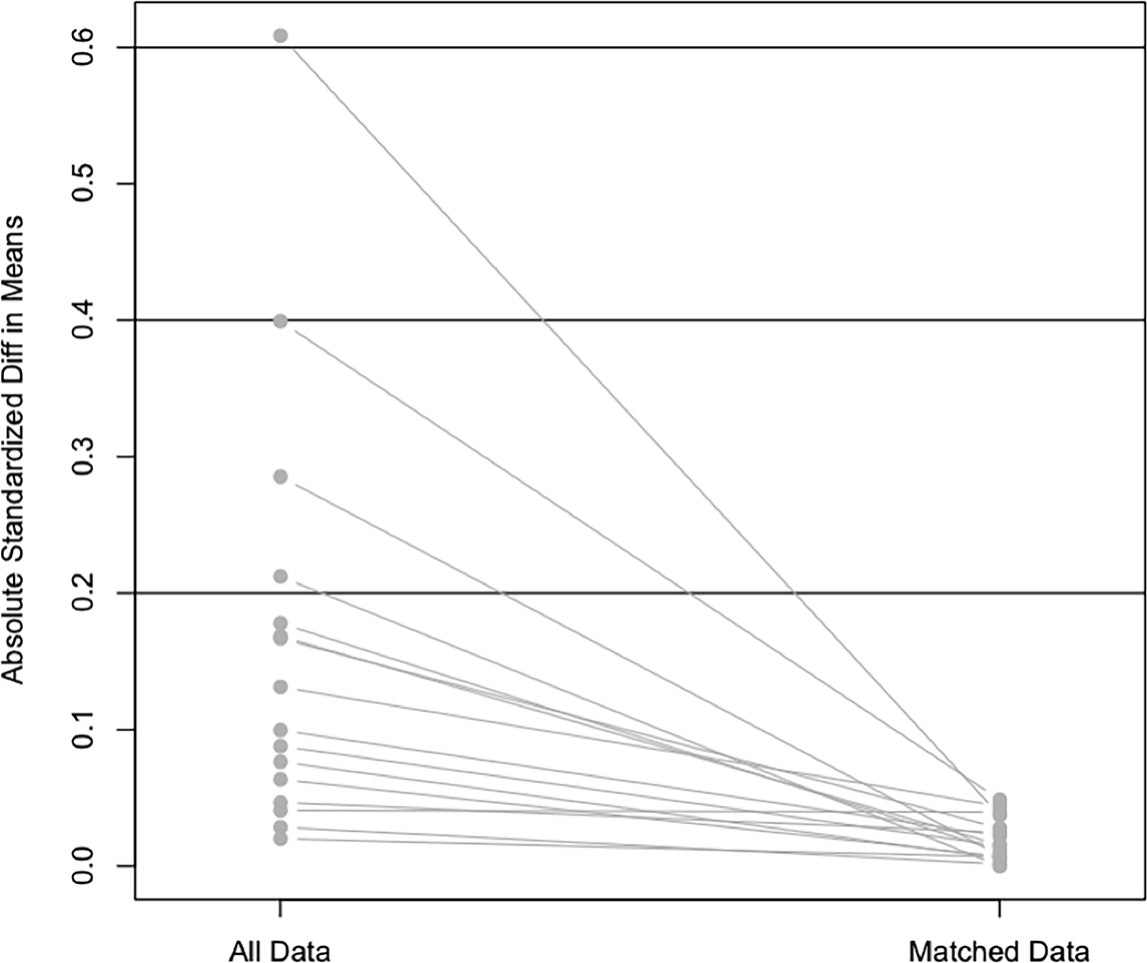
Standardized differences of means between covariates before and after match.

From December 2004 to January 2011 9,905 adult patients from 122 centers were recruited in the study. We excluded all prevalent patients and those having had less than 90 days on PD. Of the remaining 5,707 patients, we identified 1,745 who were treated exclusively by CAPD and 2,516 by APD. Of those, 1,247 who switched PD modalities, and 199 who presented missing data were excluded. Mean age was 59.4±16.0 years, 52% were females, 44% were diabetics and 36% had history of previous hemodialysis (HD). After match, 2,890 patients were included in the analysis: 1556 CAPD and 1334 APD patients (Fig 1). All variables were well balanced with the matching procedure (Table 1); the standardized differences of means between covariates can be seen in Fig 2.

**Table 1:** Clinical and demographic characteristics of matched patients.

Variable	CAPD (n = 1556)	APD (n = 1334)	*p*
**Primary Renal Disease**			0.08
Hypertension	17.6%	18.1%	
Diabetes	35.7%	37.8%	
Glomerulonephritis	9.5%	9.4%	
Other causes	18.9%	17.3%	
Unknown	18.3%	17.4%	
**Age (years)**	59.0±15.8	59.3±16.2	0.7
**Biennium**			0.9
2005/2006	27.4%	26.6%	
2007/2008	39.7%	40.6%	
2009/2010	32.9%	32.8%	
**Body Mass Index** (Kg/m^2^)	24.7±4.4	24.5±4.7	0.1
< 18.5 Kg/m^2^	5.2%	8.4%	
18.5 to 25 Kg/m^2^	52.7%	51.1%	
> 25 Kg/m^2^	42.1%	40.5%	
**Cancer** (yes)	3.1%	2.2%	0.1
**Centre Experience** (patient-year)	41.13±23.54	39.91±23.50	0.2
**Coronary Artery Disease** (yes)	20.8%	22.5%	0.3
**Davies Score**			0.6
0–1	79.1%	77.7%	
2–3	20.9%	22.3%	
**Diabetes** (yes)	43.0%	43.3%	0.9
**Education level**			1.0
≤ 4 years	30.0%	30.0%	
> 4 years	70.0%	70.0%	
**Family Income** (<2 Braz. Min.Wage)*	64.5%	64.6%	0.9
**Gender** (female)	46.0%	44.8%	0.6
**Hypertension**	77.0%	77.1%	0.9
**Peripheral Artery Disease** (yes)	20.9%	21.2%	0.9
**Race** (White)	50.3%	49.7%	0.7
**Stroke** (yes)	1.0%	1.2%	0.3
**Time of Pre-dialysis Care** (months)	18.05±30.1	17.29±29.7	0.5

* In 2006 one Brazilian minimum wage was equivalent to 128US$ and in 2010 raised to 325US$.

### Clinical outcomes

#### Technique survival

There were 344 events during the study period, 153 in the CAPD group and 191 in the APD group. Peritonitis was the main cause of technique failure in both groups representing 69.3% (n = 106) and 55.0% (n = 105), respectively for CAPD and APD. Ultrafiltration failure occurred in only 2.4% of all patients (n = 70 patients), what represents 24% (n = 47) of the technique failures in the APD group and for 15% (n = 23) in the CAPD group.

**Fig 3:**
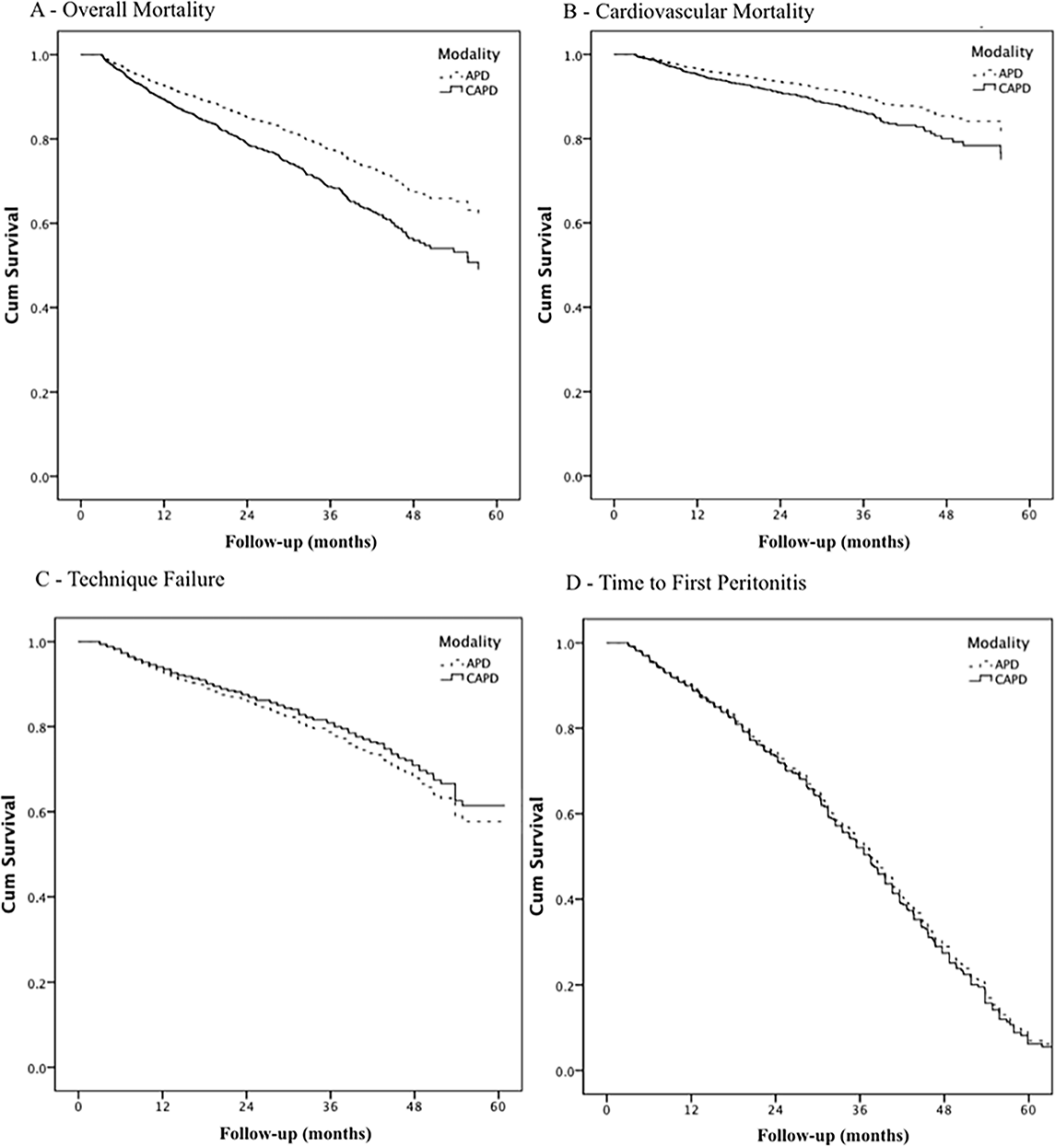
Clinical outcomes for Peritoneal Dialysis Modality. Legend: p values for overall mortality and cardiovascular mortality are < 0.01; for technique failure is 0.27 and for time to first peritonitis episode is 0.57.

In the multivariate Cox analysis, no significant difference was found between groups (HR 0.89; CI95% 0.71–1.10) (Table 2). Adjusted survival curves can be found in Fig 3. After adjustments for the presence of competing risks, the absence of differences between groups remained (SHR 0.83; CI95% 0.69–1.02). The covariates included in this model were age, year of entry in PD, BMI, center experience, diabetes, literacy, gender, race and length of pre-dialysis care. We found three independent predictors for technique failure: age, center experience and race. Details can be found in the supporting information (S1 Table).

**Table 2:** Determinants of Clinical Outcomes taking CAPD as the reference.

	Cox Model		Competing Risk Model	
	Hazard ratio	CI95%	Sub-Hazard Distribution	CI95%
Technique Failure	0.89	0.71–1.10	0.83	0.69–1.02
Time to First Peritonitis	1.04	0.90–1.20	0.96	0.93–1.11
Overall Mortality	1.47	1.24–1.75	1.44	1.21–1.71
Cardiovascular Mortality	1.41	1.09–1.82	1.34	1.03–1.73

CAPD: Continuous Ambulatory Peritoneal Dialysis; CI95% Confidence Interval 95%.

#### Time to first peritonitis episode

In the CAPD group, 368 patients had at least 1 peritonitis episode while in the APD group this number was 391. There was no difference in time to first peritonitis episode between groups in both the Cox regression analysis (HR 1.04; CI95% 0.90 to 1.20) and also considering the presence of competing risks (SHR 0.96; CI95% 0.93 to 1.11) (Table 2). For this model we included the following covariates: age, biennium, BMI, cancer, center experience, coronary artery disease, diabetes, literacy, gender, hypertension, modality, race, peripheral artery disease and length of pre-dialysis care. There were four independent predictors for time to first peritonitis episode: biennium, cancer, center experience and literacy. Full details can be found in the supporting information (S2 Table). We also analyzed peritonitis rates in both groups. For CAPD there were 0.23 episodes per patient-year and for APD 0.26 episodes per patient year.

#### Mortality

There were 550 events during the study period, 305 in the CAPD group and 245 in the APD group. Cardiovascular disease (CVD) was the main cause of death in both groups with 137 events for CAPD group (49%) and 110 events for the APD group (45%), followed by PD non-related infections (n = 104 for CAPD and n = 85 for APD), other causes (n = 38 for CAPD and n = 27 for APD), and peritonitis (n = 19 for CAPD and n = 20 for APD). In the multivariate Cox analysis, CAPD patients had a higher risk for overall (HR1.47; CI95% 1.24 to 1.75) and cardiovascular mortality (HR1.41; CI95% 1.09 to 1.82) (Table 2). Curves for Cox regression can be found in Fig 3. Results were similar when considering the presence of competing risks (SHR 1.44; CI95%1.21 to 1.71 and SHR 1.34;CI95% 1.03 to 1.73, respectively for overall and CV mortality). For both analyses, the variables included in the model were age, year of dialysis initiation, BMI, center experience, coronary artery disease, cancer, diabetes, educational level, gender, hypertension, race and length of pre-dialysis care. Age, year of initiation of PD, BMI and diabetes were independent predictors of overall mortality. Age, BMI, coronary artery disease and diabetes were independent predictors of CV mortality. Details can be found in the supporting information file: (S3 Table and S4 Table).

#### Sensitivity analysis

The group of patients without presumed RRF presented a considerably risk for mortality at univariate Cox regression model analysis and competing risk analysis (HR 1.71; CI95% 1.44–2.03 and SHR; CI9% respectively). This subgroup also presented a significant risk for technique failure (HR 1.32; CI95% 1.06–1.65) and for time to first peritonitis episode (HR 1.32; CI95% 1.13–1.53).

After inclusion of this covariate in the multivariate analysis, CAPD patients remained at high risk for mortality for all causes (SHR 1.38; CI95% 1.16–1.64) and for CV mortality (SHR 1.31; CI95% 1.01–1.69). In addition, no changes were observed for technique failure (SHR 0.82; CI95% 0.66–1.02) and time to first peritonitis (SHR 0.96; CI95% 0.83–1.10) regarding the PD modality (APD as reference).

## Discussion

This is the first large cohort study to compare hard clinical outcomes between PD modalities using a propensity match score to minimize the effect of unbalanced covariates and taking the presence of competing risks into account. The main findings of the study were that technique failure and time to first peritonitis were similar between modalities, while patient survival was better for patients who remained all the time in APD compared to those treated only with CAPD.

Peritonitis, as expected, was the main cause of technique failure[16]. Both groups presented similar time to the first peritonitis episode. It is noteworthy to mention that the connection system used by all of our patients did not change along the study period, information that is usually absent in previous reports. The twin bag system was the standard for all CAPD patients, while for APD spike connectors were utilized (luer lock connections are not available in Brazil). Despite the higher number of exchanges need for CAPD patients, time to first peritonitis episode were similar between groups. This finding is in line with previous reports from large and representative cohort studies from patients starting dialysis in early 2000[8–10], and confirm a trend that can be in part attributed to improvements in connection systems, and perhaps to an improvement in clinical practice along the years[13,17]. Similar to our results, Lan et al[3] recently showed similar overall peritonitis rates between APD and CAPD.

PD modality did not affect the rate of transfer of patients to hemodialysis in our cohort. This finding is not surprising in a population in which the main cause of technique failure was similar between groups. This scenario could have been different in a hypothetic situation, with a higher prevalence of anuric patients with high transport membrane profile. Ultrafiltration failure (UFF) occurred in only 2.4% of the patients in the present study. This is somehow expected, since the use of APD as the initial therapy in this subgroup was likely driven by factors others than the membrane profile. Instead, patients who switched from CAPD to APD (and excluded from this study) were more likely to present a higher prevalence of high transporters with a more challenging volume control. Nevertheless, information regarding membrane profile would be helpful to a better interpretation of our results.

Over the past decades reports from different regions compared patient survival between PD modalities[6–12,18]. The present study is one of the largest comparing outcomes between modalities, and in contrast to previous large cohort studies we show that patients treated with APD presented better overall and cardiovascular mortality in comparison with patients on CAPD. To the best of our knowledge, only two single center and one registry study found a benefit for patients treated by APD[6,7,12] but the present study is the first to report cardiovascular outcomes, which is the main cause of death in dialysis patients. Importantly, we matched groups for several important variables that could have influenced outcomes, including (but not restricting) age, center experience, Davies comorbidity score, diabetes, gender, literacy, coronary artery disease at baseline, previous hemodialysis etc. It is important to mention that data of some recognized risk factors were not available, such as (RRF) and the membrane profile. Nevertheless, the similar prevalence of previous hemodialysis with the exclusion of prevalent PD patients make less likely that a great difference in RFF between groups was present. In addition, our sensitivity analysis adjusted for patients with presumed RRF showed that APD patients remained with better survival rates.

**Fig 4:**
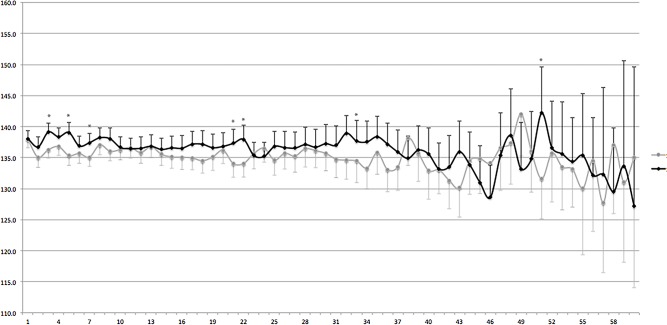
Blood pressure behavior along the study period. Legend: Markers represent mean systolic and diastolic blood pressure whilst error bars represents confidence interval 95%. The bottom box indicates the absolute number of patients included in the analysis per group.

Given the characteristics of any other observational study it is difficult to clarify the mechanisms behind the benefit of APD compared to CAPD in our population. A better volume control could be one explanation, since fluid removal is usually better achieved in APD patients, in particular the high transporters. Johnson et al reported that APD treatment was associated with a significant survival advantage in high transporters compared with CAPD[7]. In addition, we studied the behavior of blood pressure, namely systolic and diastolic blood pressure, between groups and the results advocates against this possibility since during most of the time no significant differences were found between groups (Fig 4). So, we believe that the positive effect of APD on mortality may also be related to an improvement in the management of high transporters[7]. Nevertheless, it is important to remark that the lack of data regarding peritoneal transport characteristics, sodium intake and removal, and more specifically the absence of a more specific marker of volume status, compromise any speculation in this subject.

To avoid bias potentially caused by censoring subgroups with different prognosis, we considered the presence of competing risk for this analysis as recommended by recent literature[19]. In fact, cardiovascular mortality was considerably higher in patients treated with CAPD even with similar prevalence of coronary artery disease at the beginning of the study and of others important known risk factors to CV events. The underlying mechanisms are not clear and unfortunately particular causes of the cardiovascular death are not available, an information that could have helped to speculate the mechanism behind this finding.

Finally, it is important to note that, although the selection of PD modality in many parts of the world may be driven by patient preference and economic considerations (despite the membrane profile), economic aspects were unlikely to play a role in the present cohort study, since all patients have a universal coverage of the Brazilian public healthcare system (SUS), allowing access to all PD modalities. Of note, icodextrin was not available in our country for any patient during the study period.

This study present some limitations: first, although matched and well balanced for several covariates using a propensity score approach, this method does not account for unmeasured confounders unlike a randomized controlled clinical trial; second, the particular causes of CV death were not captured in the study, and mortality related to vascular or myocardial causes cannot de differentiated; third, the absence of data related to residual renal function and membrane profile which, despite the likelihood of them being similar between groups due a similar dialysis vintage, does not exclude the possibility of there being a statistically significant difference; and finally, we were not able to retrieve data of the first peritonitis according to etiologies. However, our study has some important strengths: first, the study was based on a national prospective cohort of incident patients; the groups were well balanced for several clinical and demographic variables using sophisticated matching procedure; and finally, it is the only study of our knowledge to take competing risks into account in all analysis.

In conclusion, based in a large contemporary and prospective cohort study, no differences were found for technique failure and time to first peritonitis. In contrast, APD offered a better patient survival compared to CAPD. These findings may influence on choice of modality and may stimulate a more broad use of APD.

## Supporting Information

S1 Fig BRAZPD II structure and contacts. (TIF)

S1 Table Determinants of Overall Mortality. (DOCX)

S2 Table Determinants of Technique Failure. (DOCX)

S3 Table Determinants of Time to First Peritonitis Episode. (DOCX)

S4 Table Determinants of Cardiovascular Mortality (n = 2890). (DOCX)

S5 Table Ethical Review Boards that approved the study. (DOCX)
