# Correction: Automated Peritoneal Dialysis Is Associated with Better Survival Rates Compared to Continuous Ambulatory Peritoneal Dialysis: A Propensity Score Matching Analysis

**DOI:** 10.1371/journal.pone.0138382

**Published:** 2015-09-14

**Authors:** Gabriela de Carvalho Beduschi, Ana Elizabeth Figueiredo, Marcia Olandoski, Roberto Pecoits-Filho, Pasqual Barretti, Thyago Proenca de Moraes, on behalf of all centers that contributed to the BRAZPD

The affiliation for sixth author is incorrect. Thyago Proenca de Moraes is not affiliated with #1 but with #3 School of Medicine, Pontifícia Universidade Católica do Paraná (PUCPR), Curitiba, Brazil

The affiliation for fifth author is incorrect. Pasqual Barretti is not affiliated with #3 but with #1 School of Medicine, UNESP, Botucatu, Brazil

There is an error in Table 1. The number of patients per group is 1445 for both groups and not 1556 and 1334 as previously presented. Please see the corrected table here.

**Table 1:** Clinical and demographic characteristics of matched patients

Variable	CAPD	APD	*p*
	(n = 1445)	(n = 1445)	
**Primary Renal Disease**			0.08
Hypertension	17.6%	18.1%	
Diabetes	35.7%	37.8%	
Glomerulonephritis	9.5%	9.4%	
Other causes	18.9%	17.3%	
Unknown	18.3%	17.4%	
**Age (years)**	59.0±15.8	59.3±16.2	0.7
**Biennium**			0.9
2005/2006	27.4%	26.6%	
2007/2008	39.7%	40.6%	
2009/2010	32.9%	32.8%	
**Body Mass Index** (Kg/m^2^)	24.7±4.4	24.5±4.7	0.1
< 18.5 Kg/m^2^	5.2%	8.4%	
18.5 to 25 Kg/m^2^	52.7%	51.1%	
> 25 Kg/m^2^	42.1%	40.5%	
**Cancer** (yes)	3.1%	2.2%	0.1
**Centre Experience** (patient-year)	41.13±23.54	39.91±23.50	0.2
**Coronary Artery Disease** (yes)	20.8%	22.5%	0.3
**Davies Score**			0.6
0–1	79.1%	77.7%	
2–3	20.9%	22.3%	
**Diabetes** (yes)	43.0%	43.3%	0.9
**Education level**			1.0
≤ 4 years	30.0%	30.0%	
> 4 years	70.0%	70.0%	
**Family Income** (<2 Braz. Min.Wage)*	64.5%	64.6%	0.9
**Gender** (female)	46.0%	44.8%	0.6
**Hypertension**	77.0%	77.1%	0.9
**Peripheral Artery Disease** (yes)	20.9%	21.2%	0.9
**Race** (White)	50.3%	49.7%	0.7
**Stroke** (yes)	1.0%	1.2%	0.3
**Time of Pre-dialysis Care** (months)	18.05±30.1	17.29±29.7	0.5

* In 2006 one Brazilian minimum wage was equivalent to 128US$ and in 2010 raised to 325US$

There is an error in Table 2. Please see the corrected table here.

**Table 2:** Determinants of Clinical Outcomes taking APD as the reference

	Cox Model		Competing Risk Model	
	Hazard ratio	CI95%	Sub-Hazard Distribution	CI95%
Technique Failure	0.89	0.71–1.10	0.83	0.69–1.02
Time to First Peritonitis	1.04	0.90–1.20	0.96	0.93–1.11
Overall Mortality	1.47	1.24–1.75	1.44	1.21–1.71
Cardiovascular Mortality	1.41	1.09–1.82	1.34	1.03–1.73

CAPD: Continuous Ambulatory Peritoneal Dialysis; CI95% Confidence Interval 95%
