# Supplementary figures and images for: Automated Peritoneal Dialysis Is Associated with Better Survival Rates Compared to Continuous Ambulatory Peritoneal Dialysis: A Propensity Score Matching Analysis
Source: PLoS One. 2015 Jul 27;10(7):e0134047. doi: 10.1371/journal.pone.0134047 (PMC4516259; doi:10.1371/journal.pone.0134047)

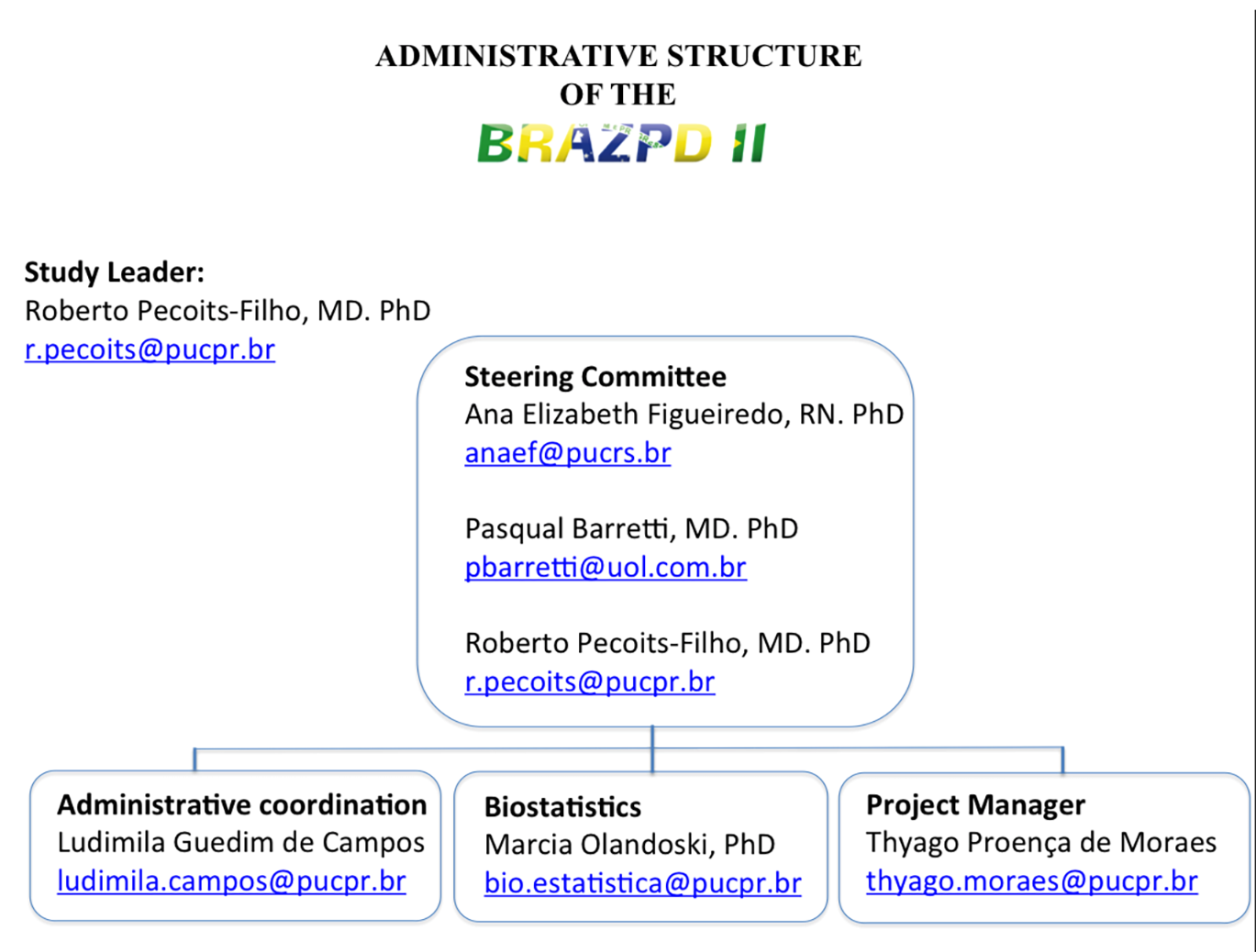

Supplement: S1 Fig — (TIF) [file pone.0134047.s001.tif]
