# Supplementary material for: Automated Peritoneal Dialysis Is Associated with Better Survival Rates Compared to Continuous Ambulatory Peritoneal Dialysis: A Propensity Score Matching Analysis
Source: PLoS One. 2015 Jul 27;10(7):e0134047. doi: 10.1371/journal.pone.0134047 (PMC4516259; doi:10.1371/journal.pone.0134047)
Supplement: S1 Table — (DOCX) [file pone.0134047.s002.docx]

**S1 Table A. Determinants of Technique Failure**

| **Model** | | | | | | | | |
| --- | --- | --- | --- | --- | --- | --- | --- | --- |
|  | **Cox** | | | | **Competing Risk** | | | |
|  | Hazard ratio | CI95% | *p* | Sub-Hazard Distribution | | | CI95% | *p* |
| Age (years) | 0.991 | 0.984-0.999 | 0.02 | | 0.986 | 0.978-0.993 | | <0.01 |
| *Biennium ^a^* |  |  |  | |  |  | |  |
| 2007/2008 | 1.02 | 0.79-1.30 | 0.9 | | 0.91 | 0.72-1.15 | | 0.4 |
| 2009/2010 | 1.04 | 0.74-1.45 | 0.8 | | 0.89 | 0.64-1.22 | | 0.4 |
| *Body Mass Index ^b^* |  |  |  | |  |  | |  |
| < 18.5 Kg/m^2^ | 0.97 | 0.62-1.52 | 0.9 | | 0.87 | 0.55-1.36 | | 0.5 |
| > 25 Kg/m^2^ | 1.16 | 0.93-1.45 | 0.2 | | 1.18 | 0.94-1.47 | | 0.1 |
| Center Experience ^c^ | 0.987 | 0.982-0.992 | <0.01 | | 0.990 | 0.984-0.995 | | <0.01 |
| Diabetes | 1.11 | 0.88-1.38 | 0.4 | | 1.02 | 0.82-1.27 | | 0.8 |
| Educational level ^d^ | 0.86 | 0.67-1.10 | 0.2 | | 0.87 | 0.68-1.12 | | 0.3 |
| Gender (female) | 1.05 | 0.85-1.31 | 0.6 | | 1.06 | 0.86-1.32 | | 0.6 |
| Modality (CAPD) | 0.89 | 0.71-1.10 | 0.3 | | 0.83 | 0.69-1.02 | | 0.08 |
| Race (White) | 0.79 | 0.63-0.98 | 0.03 | | 0.78 | 0.63-0.97 | | 0.02 |
| Pre-dialysis Care (months) | 0.996 | 0.992-1.000 | 0.05 | | 0.997 | 0.993-1.001 | | 0.1 |

CI, Confidence Interval; HD, hemodialysis; CAPD: Continuous Ambulatory Peritoneal Dialysis

^a^ Reference: patients starting dialysis in 2005/2006

^b^ Reference 18.5 to 25 Kg/m^2^

^c^ Expressed in patient-year

^d^ Reference: less than 4 years in school.
