# Supplementary material for: Automated Peritoneal Dialysis Is Associated with Better Survival Rates Compared to Continuous Ambulatory Peritoneal Dialysis: A Propensity Score Matching Analysis
Source: PLoS One. 2015 Jul 27;10(7):e0134047. doi: 10.1371/journal.pone.0134047 (PMC4516259; doi:10.1371/journal.pone.0134047)
Supplement: S2 Table — (DOCX) [file pone.0134047.s003.docx]

**S2 Table B. Determinants of Time to First Peritonitis Episode**

**Model**

|  | **Cox** | | | | **Competing Risk** | | | |
| --- | --- | --- | --- | --- | --- | --- | --- | --- |
|  | Hazard ratio | CI95% | *p* | Sub-Hazard Distribution | | | CI95% | *p* |
| Age (years) | 1.004 | 0.999-1.009 | 0.1 | | 0.998 | 0.993-1.003 | | 0.4 |
| *Biennium ^a^* |  |  |  | |  |  | |  |
| 2007/2008 | 1.37 | 1.15-1.64 | <0.01 | | 1.04 | 0.89-1.22 | | 0.6 |
| 2009/2010 | 2.44 | 1.93-3.07 | <0.01 | | 1.46 | 1.19-1.79 | | <0.01 |
| *Body Mass Index ^b^* |  |  |  | |  |  | |  |
| < 18.5 Kg/m^2^ | 1.09 | 0.81-1.45 | 0.6 | | 0.92 | 0.69-1.23 | | 0.5 |
| > 25 Kg/m^2^ | 1.03 | 0.88-1.20 | 0.7 | | 1.04 | 0.90-1.21 | | 0.5 |
| Cancer (yes) | 1.78 | 1.24-2.54 | <0.01 | | 1.67 | 1.14-2.44 | | <0.01 |
| Center Experience ^c^ | 0.991 | 0.988-995 | <0.01 | | 0.994 | 0.991-0.997 | | <0.01 |
| Coronary Artery Disease (yes) | 0.97 | 0.81-1.16 | 0.8 | | 0.96 | 0.80-1.14 | | 0.6 |
| Diabetes | 1.08 | 0.93-1.26 | 0.3 | | 0.98 | 0.95-1.13 | | 0.8 |
| Educational level ^d^ | 0.79 | 0.67-0.94 | <0.01 | | 0.75 | 0.64-0.89 | | <0.01 |
| Gender (female) | 0.97 | 0.84-1.12 | 0.6 | | 0.95 | 0.83-1.10 | | 0.5 |
| Hypertension (yes) | 0.94 | 0.79-1.12 | 0.5 | | 0.96 | 0.81-1.15 | | 0.7 |
| Modality (CAPD) | 1.04 | 0.90-1.20 | 0.6 | | 0.96 | 0.93-1.11 | | 0.6 |
| Race (White) | 0.94 | 0.81-1.09 | 0.5 | | 0.91 | 0.78-1.05 | | 0.2 |
| Peripheral Artery Disease (yes) | 1.09 | 0.91-1.30 | 0.3 | | 0.98 | 0.81-1.15 | | 0.8 |
| Pre-dialysis Care (months) | 0.997 | 0.995-0.999 | 0.03 | | 0.999 | 0.996-1.001 | | 0.3 |

CI, Confidence Interval; HD, hemodialysis; CAPD: Continuous Ambulatory Peritoneal Dialysis

^a^ Reference: patients starting dialysis in 2005/2006

^b^ Reference 18.5 to 25 Kg/m^2^

^c^ Expressed in patient-year

^d^ Reference: less than 4 years in school.
