# Supplementary material for: Automated Peritoneal Dialysis Is Associated with Better Survival Rates Compared to Continuous Ambulatory Peritoneal Dialysis: A Propensity Score Matching Analysis
Source: PLoS One. 2015 Jul 27;10(7):e0134047. doi: 10.1371/journal.pone.0134047 (PMC4516259; doi:10.1371/journal.pone.0134047)
Supplement: S3 Table — (DOCX) [file pone.0134047.s004.docx]

| **S3 Table C. Determinants of Overall Mortality**   \| **Statistical Model** \| \| \| \| \| \| \| \| --- \| --- \| --- \| --- \| --- \| --- \| --- \| \|  \| **Cox** \| \| \| **Competing Risk** \| \| \| \|  \| Hazard ratio \| CI95% \| *p* \| Sub-Hazard Distribution \| CI95% \| *p* \| \| Age (years) \| 1.04 \| 1.03-1.05 \| <0.01 \| 1.04 \| 1.03-1.05 \| <0.01 \| \| *Biennium ^a^* \|  \|  \|  \|  \|  \|  \| \| 2007/2008 \| 0.96 \| 0.80-1.15 \| 0.7 \| 0.89 \| 0.74-1.07 \| 0.2 \| \| 2009/2010 \| 0.66 \| 0.50-0.89 \| <0.01 \| 0.57 \| 0.43-0.76 \| <0.01 \| \| *Body Mass Index ^b^* \|  \|  \|  \|  \|  \|  \| \| < 18.5 Kg/m^2^ \| 1.42 \| 1.05-1.92 \| 0.02 \| 1.42 \| 1.04-1.94 \| 0.03 \| \| > 25 Kg/m^2^ \| 0.89 \| 0.74-1.06 \| 0.2 \| 0.87 \| 0.73-1.05 \| 0.1 \| \| Center Experience ^c^ \| 0.994 \| 0.990-0.997 \| <0.01 \| 0.997 \| 0.993-1.000 \| 0.08 \| \| CAD (yes) \| 1.13 \| 0.93-1.36 \| 0.2 \| 1.14 \| 0.95-1.38 \| 0.1 \| \| Cancer (yes) \| 1.20 \| 0.76-1.88 \| 0.4 \| 1.20 \| 0.78-1.85 \| 0.4 \| \| Diabetes \| 1.49 \| 1.25-1.77 \| <0.01 \| 1.46 \| 1.22-1.73 \| <0.01 \| \| Educational level ^d^ \| 0.93 \| 0.76-1.15 \| 0.5 \| 0.91 \| 0.74-1.12 \| 0.4 \| \| Gender (female) \| 0.91 \| 0.77-1.09 \| 0.3 \| 0.90 \| 0.76-1.07 \| 0.2 \| \| Hypertension (yes) \| 0.98 \| 0.80-1.20 \| 0.9 \| 0.99 \| 0.81-1.22 \| 0.9 \| \| Modality (CAPD) \| 1.47 \| 1.24-1.75 \| <0.01 \| 1.44 \| 1.21-1.71 \| <0.01 \| \| Race (White) \| 1.11 \| 0.93-1.33 \| 0.2 \| 1.17 \| 0.97-1.39 \| 0.09 \| \| Pre-dialysis Care (months) \| 0.997 \| 0.994-1.000 \| 0.06 \| 0.998 \| 0.994-1.001 \| 0.1 \|   CI, Confidence Interval; HD, hemodialysis; CAPD: Continuous Ambulatory Peritoneal Dialysis; CAD: Coronary Artery Disease  ^a^ Reference: patients starting dialysis in 2005/2006  ^b^ Reference 18.5 to 25 Kg/m^2^  ^c^ Expressed in patient-year  ^d^ Reference: less than 4 years in school. |
| --- | --- | --- | --- | --- | --- | --- | --- | --- | --- | --- | --- | --- | --- | --- | --- | --- | --- | --- | --- | --- | --- | --- | --- | --- | --- | --- | --- | --- | --- | --- | --- | --- | --- | --- | --- | --- | --- | --- | --- | --- | --- | --- | --- | --- | --- | --- | --- | --- | --- | --- | --- | --- | --- | --- | --- | --- | --- | --- | --- | --- | --- | --- | --- | --- | --- | --- | --- | --- | --- | --- | --- | --- | --- | --- | --- | --- | --- | --- | --- | --- | --- | --- | --- | --- | --- | --- | --- | --- | --- | --- | --- | --- | --- | --- | --- | --- | --- | --- | --- | --- | --- | --- | --- | --- | --- | --- | --- | --- | --- | --- | --- | --- | --- | --- | --- | --- | --- | --- | --- | --- | --- | --- | --- | --- | --- | --- | --- | --- | --- | --- | --- | --- | --- | --- | --- | --- | --- | --- | --- | --- |
