# Supplementary material for: Automated Peritoneal Dialysis Is Associated with Better Survival Rates Compared to Continuous Ambulatory Peritoneal Dialysis: A Propensity Score Matching Analysis
Source: PLoS One. 2015 Jul 27;10(7):e0134047. doi: 10.1371/journal.pone.0134047 (PMC4516259; doi:10.1371/journal.pone.0134047)
Supplement: S4 Table — (DOCX) [file pone.0134047.s005.docx]

| **S4 Table D. Determinants of Cardiovascular Mortality (n=2890)** | | | | | | | | |
| --- | --- | --- | --- | --- | --- | --- | --- | --- |
| **Model** | | | | | | | | |
|  | **Cox** | | | | **Competing Risk** | | | |
|  | Hazard ratio | CI95% | *p* | Sub-Hazard Distribution | | | CI95% | *p* |
| Age (years) | 1.04 | 1.03-1.05 | <0.01 | | 1.036 | 1.026-1.047 | | <0.01 |
| *Biennium ^a^* |  |  |  | |  |  | |  |
| 2007/2008 | 0.91 | 0.69-1.20 | 0.5 | | 0.85 | 0.65-1.12 | | 0.2 |
| 2009/2010 | 0.59 | 0.38-0.91 | 0.02 | | 0.50 | 0.33-0.77 | | <0.01 |
| *Body Mass Index ^b^* |  |  |  | |  |  | |  |
| < 18.5 Kg/m^2^ | 1.04 | 0.62-1.74 | 0.9 | | 0.98 | 0.58-1.66 | | 0.1 |
| > 25 Kg/m^2^ | 0.97 | 0.74-1.26 | 0.8 | | 0.99 | 0.76-1.29 | | 0.1 |
| Cancer (yes) | 1.51 | 0.80-2.88 | 0.2 | | 1.52 | 0.82-2.79 | | 0.2 |
| Center Experience ^c^ | 0.994 | 0.988-1.000 | 0.05 | | 0.997 | 0.991-1.002 | | 0.2 |
| Coronary Artery Disease (yes) | 1.56 | 1.19-2.03 | <0.01 | | 1.62 | 1.23-2.14 | | <0.01 |
| Diabetes | 1.44 | 1.11-1.87 | <0.01 | | 1.43 | 1.11-1.85 | | <0.01 |
| Educational level ^d^ | 0.90 | 0.66-1.22 | 0.5 | | 0.86 | 0.63-1.17 | | 0.3 |
| Gender (female) | 0.98 | 0.76-1.27 | 0.9 | | 1.02 | 0.79-1.31 | | 0.9 |
| Hypertension (yes) | 0.99 | 0.73-1.35 | 0.1 | | 1.04 | 0.76-1.42 | | 0.8 |
| Modality (CAPD) | 1.41 | 1.09-1.82 | <0.01 | | 1.34 | 1.03-1.73 | | 0.03 |
| Race (White) | 1.10 | 0.84-1.44 | 0.5 | | 1.08 | 0.82-1.41 | | 0.6 |
| Peripheral Artery Disease (yes) | 1.28 | 0.97-1.70 | 0.08 | | 1.16 | 0.86-1.55 | | 0.3 |
| Pre-dialysis Care (months) | 0.995 | 0.990-1.000 | 0.05 | | 0.996 | 0.991-1.001 | | 0.1 |

CI, Confidence Interval; HD, hemodialysis; CAPD: Continuous Ambulatory Peritoneal Dialysis

^a^ Reference: patients starting dialysis in 2005/2006

^b^ Reference 18.5 to 25 Kg/m^2^

^c^ Expressed in patient-year

^d^ Reference: less than 4 years in school.
