# Supplementary material for: Automated Peritoneal Dialysis Is Associated with Better Survival Rates Compared to Continuous Ambulatory Peritoneal Dialysis: A Propensity Score Matching Analysis
Source: PLoS One. 2015 Jul 27;10(7):e0134047. doi: 10.1371/journal.pone.0134047 (PMC4516259; doi:10.1371/journal.pone.0134047)
Supplement: S5 Table — (DOCX) [file pone.0134047.s006.docx]

**Table S5.** Ethic Review Boards that approved the study

| Ameneg - Assistência Médica Nefrológica de Guarulhos |
| --- |
| Associaçao Hospital de Bauru |
| Biocor - Hospital de Doencas Cardiologicas |
| Casa de Saude e Maternidade Nossa Senhora do Perpétuo Socorro |
| Clínica de Doenças Renais - Curitiba |
| Clínica de Doenças Renais - Goiania |
| Clínica de Doenças Renais - Imperatriz |
| Clínica de Doenças Renais - Sao Jose Pinhais |
| CDTR_Centro Dialise Transplante Renal |
| Centro Nefrologia Teresopolis |
| Centro Nefrologico Minas Gerais |
| Centro Trat. Doencas Renais Joinville |
| Centro Tratamento Renal Zona Sul |
| Clinica de Nefrologia Santa Teresa - Rio De Janeiro |
| CLINEPA Clinica de Nefrologia da Paraiba |
| Clines |
| Clinica de Nefrologia do Sergipe - CLINESE |
| Clinica do Rim do Carpina |
| Clinica Evangelico S/C Ltda |
| Clinica Nefrologia de Franca |
| Clinica de Nefrologia Santa Rita |
| Clinica de Nefrologica Sao Goncalo |
| Clinica Paulista de Nefrologia |
| Clinica Renal Manaus |
| Clinica Senhor Do Bonfim |
| Clinica Senhor Do Bonfim Ltda Filial |
| Clinica Tratamento Renal |
